# Transcriptome of the pygmy grasshopper *Formosatettix qinlingensis* (Orthoptera: Tetrigidae)

**DOI:** 10.7717/peerj.15123

**Published:** 2023-03-30

**Authors:** Yuxin Liu, Xuejuan Li, Liliang Lin

**Affiliations:** Shaanxi Normal University, Xi’an, China

**Keywords:** Tetriginae, Pigment, Immunity, Insecticide resistance

## Abstract

*Formosatettix qinlingensis* (Zheng, 1982) is a tiny grasshopper endemic to Qinling in China. For further study of its transcriptomic features, we obtained RNA-Seq data by Illumina HiSeq X Ten sequencing platform. Firstly, transcriptomic analysis showed that transcriptome read numbers of two female and one male samples were 25,043,314, 24,429,905, and 25,034,457, respectively. We assembled 65,977 unigenes, their average length was 1,072.09 bp, and the length of N50 was 2,031 bp. The average lengths of *F. qinlingensis* female and male unigenes were 911.30 bp, and 941.82 bp, and the N50 lengths were 1,745 bp and 1,735 bp, respectively. Eight databases were used to annotate the functions of unigenes, and 23,268 functional unigenes were obtained. Besides, we also studied the body color, immunity and insecticide resistance of *F. qinlingensis.* Thirty-nine pigment-related genes were annotated. Some immunity genes and signaling pathways were found, such as JAK-STAT and Toll-LIKE receptor signaling pathways. There are also some insecticide resistance genes and signal pathways, like nAChR, GST and DDT. Further, some of these genes were differentially expressed in female and male samples, including pigment, immunity and insecticide resistance. The transcriptomic study of *F. qinlingensis* will provide data reference for gene prediction and molecular expression study of other Tetrigidae species in the future. Differential genetic screening of males and females provides a basis for studying sex and immune balance in insects.

## Introduction

With the development of high-throughput sequencing technology, gene sequencing has become more accurate, cheaper, and faster. It is increasingly popular to study the gene expression of non-model species by measuring transcriptome data, which is used to find differentially expressed genes (DEGs) to verify the biological phenomena. Directly studying transcribed expressed genes can be the most intuitive and effective way to explore the expression of genes, infer the function of genes, and reveal the mechanism of gene regulation (Kjer et al., 2016). In insect research, transcriptome data is a good choice for exploring insect gene expression due to the difficulty of genome assembly and significant differences in gene numbers. For example, the genome size of *Lymantria dispar* is only 998 Mb, but that of *Locusta migratoria* is as high as 6.5 Gb (Sparks et al., 2021; Wang et al., 2014). At present, transcriptomic data of several Orthoptera insects have been analyzed, such as *Gryllus bimaculatus* (Kono et al., 2021), *Gryllus firmus* (Nanoth Vellichirammal et al., 2014), *Epacromius coerulipes* (Jin et al., 2016), *Oedaleus asiaticus* (Huang et al., 2017), *Xenocatantops brachycerus* (Zhao et al., 2018), *Ceracris nigricornis* Walker (Yuan et al., 2019), *Acheta domesticus* (Oppert et al., 2020), *Calliptamus italicus* (Liu et al., 2022).

*Formosatettix qinlingensis* (Zheng, 1982) is a tiny grasshopper endemic to China, almost exclusively distributed in Qinling Mountains (Shaanxi, Henan and Gansu). *Formosatettix qinlingensis* usually has a small body size, with males 10–10.5 mm long and females 12.5–13 mm long (Zheng, 2005). Grasshoppers are very sensitive to environmental changes (Leonard et al., 2021). The study of *F. qinlingensis* can be used as an indicator for natural environment analysis, and can also be used to biological control grasshoppers in the field. Exploring the gene expression of *F. qinlingensis* provides a template for studying body color, immunity defense, and insecticide resistance of grasshoppers. So far, due to small size and limited distribution, the physiological, ecological and genomic studies of the *F. qinlingensis* were limited, which requires more in-depth research. Among them, Lin et al. (2017) assembled and analyzed the mitochondrial genome of *F. qinlingensis*.

Insect body color has always been a hot topic in the study of insects, and has a wide range of values in insect morphology, physiology, ecology, development, genetics and molecular biology (Wittkopp, Carroll & Kopp, 2003; Futahashi et al., 2012; Seike & Nagata, 2021). Transcriptome research is an excellent method to study pigment differences because it can directly target the genes or transcriptional components that lead to body color differences (Wittkopp, Carroll & Kopp, 2003). For example, the neuropeptide [His7]-corazonin is a dark color–inducing factor for *L. migratoria* (Tanaka, 2001).

With the development of agriculture, the spraying of various pesticides has a significant impact on the living environment of insects. Pesticide residues exist in farmland, water, atmosphere and plants (Koçyiğit & Sinanoğlu, 2020). A large number of pesticide residues require that insects have the ability to resist pesticides. Common insecticides in the market include organophosphate, pyrethroid, neonicotinoid, carbamate, cyclodiene and pyrazole, diamide, *etc*. They kill insects through different metabolic pathways and target receptors. For example, cyclodiene and pyrazole insecticides act on *γ*-amino butyric acid (GABA), which is an inhibitory neurotransmitter of insects, can block chloride channels to kill insects. The target of neonicotinoid insecticides is the acetylcholine receptor (nAChR), which is the primary excitatory receptor in the central nervous system of insects. Neonicotinoid insecticides have a higher ability to kill insects, and also have a solid ability to kill eggs and larvae. The mechanisms of insect response to insecticides have been studied in several species, including Lepidoptera (*Spodoptera frugiperda, Manduca sexta*), Blattodea (*Nauphoeta cinerea, Periplaneta americana*), Diptera (*Aedes aegypti, Drosophila melangaster*), Hemiptera (*Dysdercus peruvianus, Oncopeltus fasciatus, Rhodnius prolixus, Triatoma infestans*), Orthoptera (*L. migratoria, Schistocerca americana*) (Carlini et al., 1997; Barreto et al., 2020). The body color of *F. qinlingensis* is mainly brown, which is similar to living environment, the color of soil and rock. Such color can make them avoid natural enemies effectively, but it can’t make them avoid pesticides. Resistance to insecticides, as innate immune and acquired immune mechanisms have become indispensable factors for their survival.

This study assembled and annotated the transcriptome data of female and male *F. qinlingensis* samples, and screened the genes and signal pathways related to pigment, immunity and insecticide resistance. Based on annotation, the genes and pathways differentially expressed by both sexes were screened. This provides a molecular basis for the subsequent development of gene function annotations, immunity genes or pathways, insecticide resistance genes, and molecular markers of *F. qinlingensis*. As limited transcriptomic study were carried out, this may also provide data reference for gene prediction and molecular expression study of other Tetrigidae species in the future.

## Materials and Methods

### Species collection and sample preparation

*F. qinlingensis* was collected in Xi’an, Shaanxi in 2017, including adult samples of three male individuals combined into one sample and six female individuals formed as two samples. Species identification follows Zheng (2005) and Deng et al. (2023) (Fig. S1). In order to ensure the acquisition of transcriptome data for small insects, three individuals were mixed as one sample. Three samples of *F. qinlingensis* were used for sequencing, in which females had two repeats. The RNAs were obtained using the RNA extraction kit (Trizol), and sequencing was carried out based on the Illumina HiSeq X Ten sequencing platform. To ensure that the sample can meet the transcriptome sequencing standard, Nanodrop was used to detect the purity of the sample, Qubit 2.0 was used to measure the sample concentration, and Agilent 2100 was used to detect the RNA integrity.

After passing the inspection, eukaryotic mRNA was enriched by Oligo (dT) magnetic beads, and then the purified mRNA was fragmented by fragmentation reagent (AM8740, Invitrogen). The first cDNA strand used mRNA as a template and was synthesized by adding random hexamers. The second strand was synthesized by adding buffer, dNTPs, RNase H, and DNA polymerase I. cDNA was purified using AMPure XP Beads. Finally, the cDNA library was obtained by terminal repair, A-tail addition and sequencing adaptor attachment, fragment size selection, and PCR amplification.

The obtained cDNA library was subject to quality testing. Qubit 2.0 was used to measure the cDNA concentration, Agilent 2100 was used to determine the insert size, and Q-PCR was used to determine the effective concentration. After passing the test, the transcriptomic libraries of female and male *F. qinlingensis* were sequenced by Illumina HiSeq X Ten. The transcriptomic data of *F. qinlingensis* has been stored in the NCBI database with the accession numbers of SRR19977122, SRR19977123, and SRR19977124.

### *De novo* assembly and assembly completeness assessment

Trinity v2.5.1 was used to break sequencing reads into smaller fragments (K-mer) (Grabherr et al., 2011). The transcripts and unigenes were obtained from assemble results. The completeness of transcriptome assembly was evaluated by BUSCO v5.4.3 (Waterhouse et al., 2018).

### Functional annotation

This study compared the transcriptome data with multiple databases to obtain more comprehensive functional annotation information. BLAST was used to compare unigenes with NR, Swiss-Prot, GO, COG, KOG, eggNOG4.5 and KEGG databases to obtain annotation results of each database. Then KOBAS2.0 was used to obtain KEGG Orthology results for unigenes. Finally, using the software Hmmer v3.1b2, the predicted unigene amino acid sequences were compared with Pfam database to complete the functional annotation of transcriptional sequences. During the annotation process, we selected the BLAST *E*-value not greater than 1e−5 and a Hmmer parameter *E*-value not greater than 1e−10.

### Differential expression of unigenes (DEUs)

The power analysis calculation was performed using the RNASeqPower v3.15 in RGui package v4.2.0 (Hart et al., 2013). The statistical power was 0.9595131, with parameter settings of depth = 20, *n* = 3, CV = 0.4, effect = 4, alpha = 0.05). Sufficient effective data was a key condition for accurate information analysis. The detection results of FPKM method were used to evaluate data saturation, which proves that sufficient data amount had been obtained in the experiment. The significance threshold of DEUs identification was selected as both FDR (False Discovery Rate) < 0.01 and FC (fold change) ≥ 2.

GO enrichment and KEGG enrichment were mainly used to annotate DEUs, and COG and KOG were used to assist in the analysis of differences. KEGG calculated the enrichment factor by following formula, and used Fisher to calculate the enrichment significance to analyze the degree of pathway enrichment (Poirel, Owens & Murali, 2011).

\begin{eqnarray*}\text{Enrichment}~\text{Factor}= \frac{\text{Pathway}~\text{DEUs}/\mathrm{All}~\text{DEUs}}{\text{Pathway}~\text{genes}/\text{KEGG}~\mathrm{all}~\text{genes}} . \end{eqnarray*}

### Detection of SNPs and SSRs

The software STAR v2.6.0b aligns the sequences between reads and unigenes (Dobin et al., 2013). The Single Nucleotide Polymorphism (SNP) sites were recognized through the GATK v3.2.2 recognition process (McKenna et al., 2010).

MISA (http://pgrc.ipk-gatersleben.de/misa/) identifies six types of simple sequence repeat (SSR) by analyzing unigenes of more than 1 kb (Beier et al., 2017). It included mono-nucleotide repeat, di-nucleotide repeat, tri-nucleotide repeat, tetra-nucleotide repeat, penta-nucleotide repeat and hexa-nucleotide repeat.

### Quantitative real-time RT–PCR (qRT–PCR) analysis

qRT–PCR verifies the reliability of sequencing analysis results. Using the same RNA samples that were sequenced, cDNAs from male and female samples were reverse transcribed according to the FastKING RT Kit (KR180123, TianGen) instructions. Differentially expressed genes were selected to design primers. The PCR program used included denaturation at 95 °C for 15min, followed by a two-step reaction protocol of 40 cycles of 95 °C for 10 s and 60 °C for 30 s. qRT-PCR had a reaction volume of 20 µL, including 10 µL SuperReal PreMix plus, 0.6 µL forward primers, 0.6µL reverse primers (Table S1), 2 µL cDNA, 0.5 µL ROX Reference Dye and 6.3µL RNase-free dd H_2_O. Each unigene was calculated using the 2- ΔΔCt formula, and *β*-actin (housekeeping gene) was used as the internal reference.

## Results

### Transcriptome sequencing, *de novo* assembly and quality assessment

The female and male samples of *F. qinlingensis* generated clean data of 7,496,972,582, 7,315,066,702 and 7,485,519,406 bp, respectively, of which the pair-end reads are 25,043,314, 24,429,905 and 25,034,457. The percentage of bp with quality scores greater or equal to Q30 were 92.49%, 93.05%, and 92.92%, and the GC content was 46.26%, 46.05%, and 45.99%.

Female samples obtained 131,436 transcripts, and males obtained 126,806 transcripts. Female samples expressed 50,199 unigenes, accounting for 76.09% of all transcriptome data, and males expressed 50,978 unigenes, accounting for 77.27% of all transcriptome data. Among them, 15,778 unigenes (23.91%) were only expressed in females, and 14,999 unigenes (22.73%) were only expressed in males. In total, 65,977 unigenes were obtained by combining female and male samples, with an average length of 1,072.09 bp and N50 length of 2,031 bp. Unigenes lengths range from 200 bp to 17,066 bp. The average lengths of *F. qinlingensis* female and male unigenes are 911.30 bp and 941.82 bp, respectively, and the N50 length are 1,745 bp and 1,735 bp (Table 1).

BUSCO v5.4.3 was used to evaluate the completeness from the assembly results using the insecta odb10 database. The integrity and accuracy of the assembly data were evaluated according to the results of the alignment between the assembly sequence and the conservative sequence in the OrthoDB database (Waterhouse et al., 2018). The results showed that the complete BUSCO score of female and male sample was 96.6%, with corresponding value 88.5% of female samples and 87.8% of male samples, respectively, which showed that the assembled *F. qinlingensis* genome sequence was complete.

### Functional annotation

**Figure 1:**
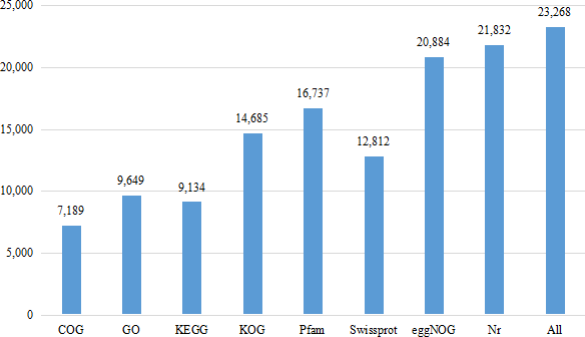
Number of unigenes annotated with Nr, Swiss-Prot, GO, COG, KOG, eggNOG, KEGG and Pfam databases of *Formosatettix qinlingensis* transcriptome data.

Eight databases were used to complete the functional annotation of transcriptome data, and 23,268 unigenes were annotated (Fig. 1). The Nr database is a non-redundant protein database in NCBI (Bagheri, Severin & Rajan, 2020). Compared with BLASTX database (Yang, Jiang & Zhang, 2014), 21,832 unigenes were annotated, accounting for 33.09% of the total data. 44,145 unigenes were not annotated, accounting for 66.91% of the total data.

**Table 1:** Statistics of *Formosatettix qinlingensis* transcriptome data.

	Female samples		Male sample	All Unigenes
	Female sample 1	Female sample 2		
Clean data	7,496,972,582	7,315,066,702	7,485,519,406	–
Pair-end reads	25,043,314	24,429,905	25,034,457	–
Q30	92.49%	93.05%	92.92%	–
GC content	46.26%	46.05%	45.99%	–
Total unigenes	50,199		50,978	65,977
Total transcripts	131,436		126,806	–
Total length	45,746,400		48,011,163	70,733,489
N50 length	1,745		1,735	2,031
Mean length	911.3		941.8	1,072.09

**Figure 2:**
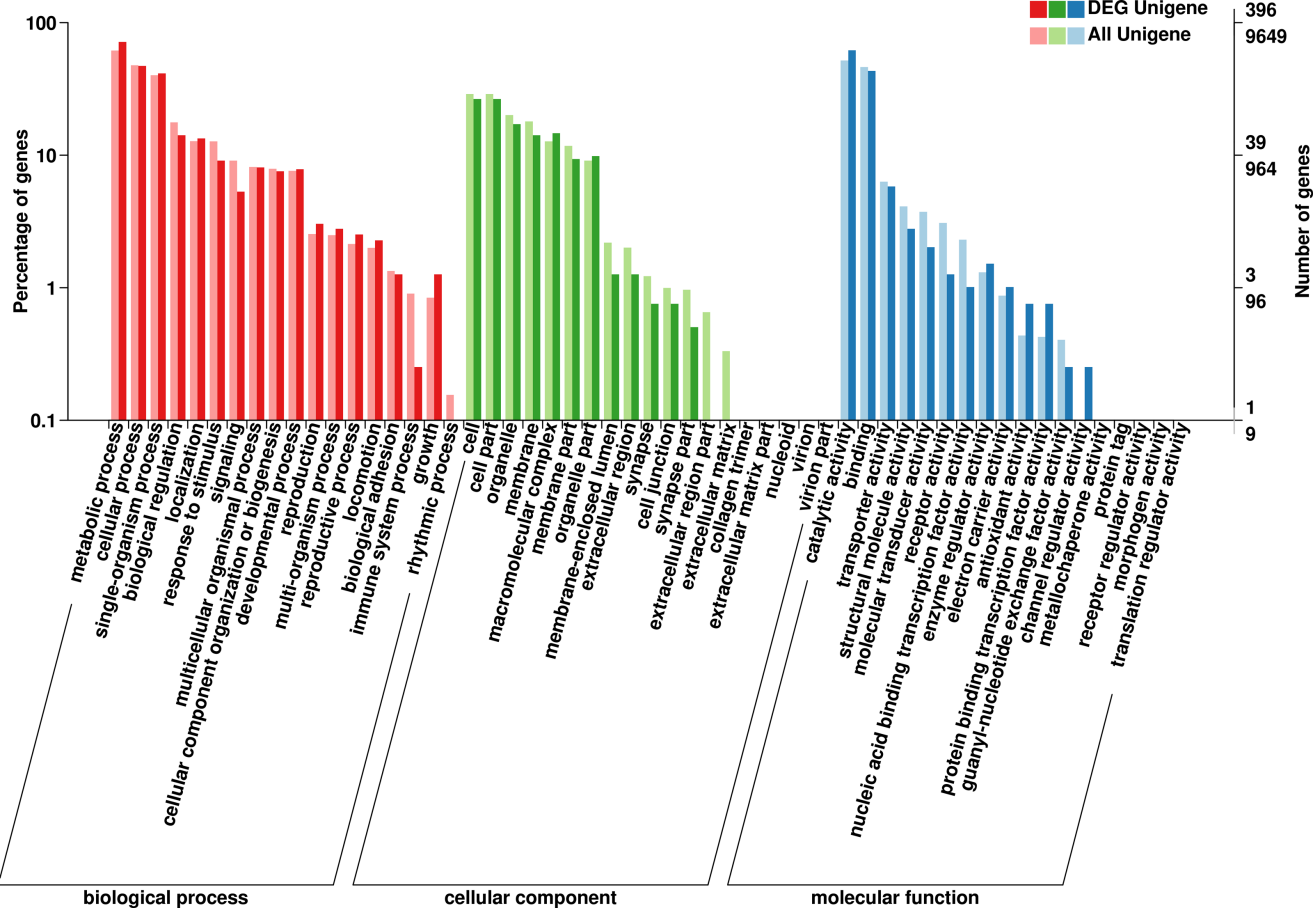
GO annotations on *Formosatettix qinlingensis*.

The GO database has a standard classification system, which divides genes and gene products into three types (molecular function, cellular component and biological process). *F. qinlingensis* annotated 9,649 unigenes, accounting for 14.62% of all unigenes (Fig. 2). In molecular function, ‘catalytic activity’ (4,993, 51.75%) and ‘binding’ (4,467, 46.29%) play an important role. In cellular component, the expression of ‘cell’ (2,790, 28.91%), ‘cell part’ (2,790, 28.91%), ‘organelle’ (1,937, 20.07%), ‘membrane’ (1,733, 17.96%), ‘macromolecular complex’ (1,225, 12.70%), ‘membrane part’ (1,134, 11.75%), and ‘organelle part’ (877, 9.09%) are relatively high. In biological process, ‘metabolic process’ (5,941, 61.57%), ‘cellular process’ (4,609, 47.77%) and ‘single-organism process’ (3,877, 40.18%) are more prominent. ‘Cell part’ and ‘catalytic activity’ are also highly expressed in *T. japonica* (Qiu et al., 2017).

 COG is a database for homologous classification of gene expression products by comparing protein sequences and analyzing protein homology. The transcriptional data were homologous to 7,189 unigenes. The results showed that the highest ranking was ‘general function prediction only’, which was annotated to 2,296 unigenes, accounting for 31.94% of all annotations, which is much higher than the other homologous genes. The remaining accounted for more are ‘replication, recombinaton and repair’ (909, 12.64%), ‘amino acid transport and metabolism’ (723, 10.06%), ‘transcription’ (645, 8.97%), ‘translation, ribosomal structure and biogenesis’ (621, 8.39%).

A total of 9,134 unigenes were annotated in the KEGG database. They were distributed in 269 pathways, such as JAK-STAT signaling pathway, Toll-LIKE signaling pathway, Wnt signaling pathway, P450 signaling pathway, RyR signaling pathway and GABA signaling pathway. Among all the pathways, ribosome (258, ko03010), purine metabolism (219, ko00230), protein processing in endoplasmic reticulum (212, ko04141), carbon metabolism (200, ko01200) had more unigenes.

### DEUs analyses

**Figure 3:**
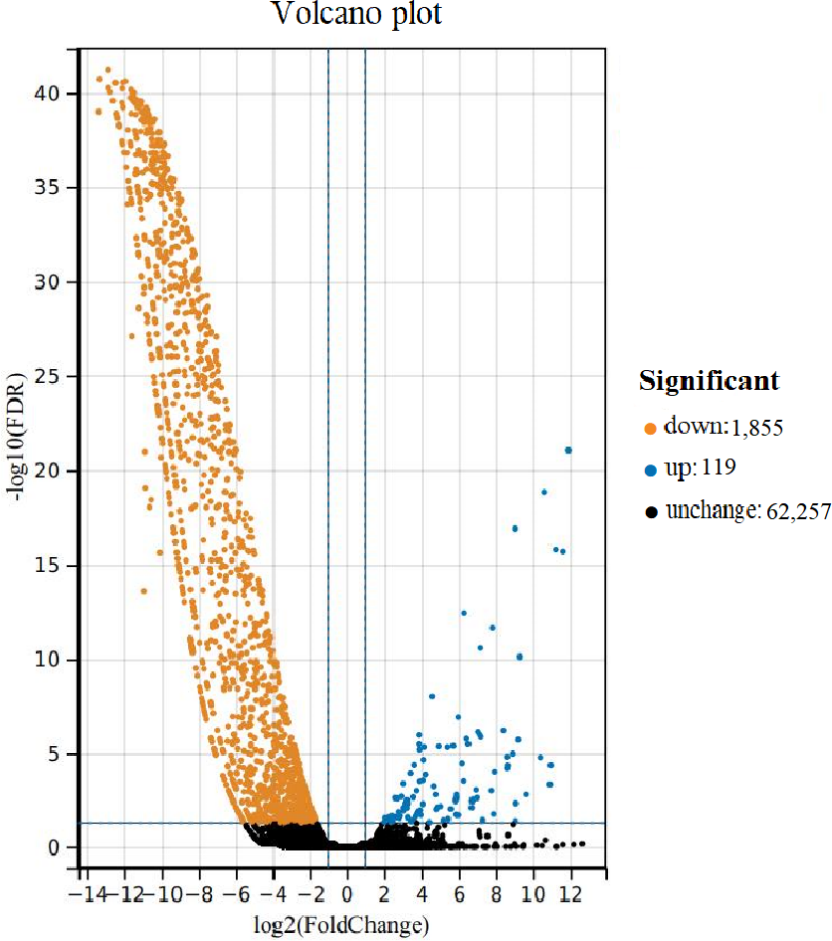
Volcano plot of differentially expressed unigenes (DEUs) for *Formosatettix qinlingensis*.

Comparing the DEUs of female and male samples, 1,974 unigenes were differentially expressed. The expression of male *vs* female samples contained 1,855 down-regulated unigenes and 119 up-regulated unigenes (Fig. 3). The DEUs were annotated by Nr, Swiss-Prot, GO, COG, KOG, eggNOG4.5 and KEGG databases, with 1,213, 719, 396, 413, 801, 1,143 and 567 unigenes, respectively.

 In the annotations of the GO database,the top four differentially expressed molecular functions were ‘catalytic activity’, ‘binding, transporter activity’, ‘structural molecule activity’ and ‘enzyme regulator activity’. Significant differences in cellular component were ‘cell’, ‘cell part’, ‘organelle, membrane’, ‘macromolecular complex’, ‘membrane part’ and ‘organelle part’. In biological process, ‘metabolic process’, ‘cellular process’ and ‘single-organism process’ occupy prominent positions.

Relevant pathways were found for 567 unigenes in the KEGG database. All mapped pathways include ‘cellular process’, ‘environmental information processing’, ‘genetic information process’ and ‘metabolism’, in which ‘metabolism’ accounts for the highest proportion with 44.97%. Among all relevant pathways, ‘carbon metabolism’ (ko01200), ‘purine metabolism’ (ko00230), ‘ubiquitin mediated’ proteolysis (ko04120), ‘glycolysis/gluconeogenesis’ (ko00010) and ‘biosynthesis of amino acids’ (ko01230) were annotated to most unigenes. Enrichment analysis was performed on the KEGG pathways annotated by DEUs, and the top 20 enrichment results were listed in Fig. S2.

### qRT-PCR results

The DEGs of male *vs* female samples involved in Wnt signaling pathway, ommochrome and bric-à-brac loci were verified by qRT-PCR experimental results. Among them, Wnt signaling pathway plays an immunoregulatory role in *F. qinlingensis*, with PKA (K04345), Siah-1 (K04506) and Cull (K03347) differentially expressed. The expression of ap3s1 of ommochrome and bric-à-brac 2 protein were significantly different, and they were related to the expression of pigment(Table S2). Overall, consistent expression patterns for selected unigenes were produced between the qRT-PCR results and RNA-Seq analyses (Fig. S3).

### Candidate genes involved in pigment

The genes related to pigment expression related genes obtained from all transcripts mainly include pteridine, melanin, ommochrome, dopamine, optomotor-blind (omb), hedgehog (Hh), bric-à-brac (bab), doublesex, and decapentaplegic (Dpp). A total of 39 unigenes were detected, details shown in Table S2. *F. qinlingensis* has a variety of body color changes, which depend on the joint regulation of a variety of pigment related genes.

Among the 39 pigment-related genes, three DEGs in female and male samples were found. Differentially expressed pigments were ommochrome and bab, both of which were significantly down-regulated in males (Table S2). In *F. qinlingensis*, ommochrome annotated four genes, and only one gene ap3s1 was down-regulated in males. *F. qinlingensis* annotated 11 bab genes, of which two genes of bab1 and bab2 were differentially expressed in females. This is consistent with the study on the mechanism of bab expression in *D. melanogaster*. The expression of various pigment-regulated genes explains its body color, which close to the living environment, and also provided a good material for studying other insect body color.

### Immunity genes and pathways

The immune-related components and pathways in the transcriptome data of *F. qinlingensis* were screened, and 13 main immunity genes were identified, including C-type lectins (CTL) (2), superoxide dismutases (SOD) (2), peptidoglycan recognition protein (PGRP) (4), apolipophorin (5) (Table S2). In addition, JAK-STAT signaling pathway (ko04630), Toll-LIKE receptor signaling pathway (ko04620) and Wnt signaling pathway (ko04310) play an immunoregulatory role in *F. qinlingensis*.

Among all selected immunity genes and pathways, JAK-STAT signaling pathway and Wnt signaling pathway are differentially expressed pathways (DEPs) in *F. qinlingensis* sex. In the JAK-STAT signal pathway, PIAS and CBP were down-regulated in males compared with females. In the Wnt signaling pathway, the expressions of PKA, Siah-1, Cull, Stbm, CaN and CBP were lower in males.

### Insecticide resistance genes and pathways

The antagonistic relationship between insects and pesticides has been a topic of debate. Related genes carboxylesterase (Car Es) (94), glutathione s-transferase(GST) (62), nicotinic acetylcholine receptor (nAChR) (25) and DDT (4) were found in the transcriptome data of *F. qinlingensis*. There are also some related signaling pathways, including GABA signal pathway (ko05034), RyR signal pathway (ko04961, ko04962) and P450 signal pathway (ko00980, ko00982). Among them, only P450 signaling pathway contains DEPs (ko00980, ko00982).

### Identification of SNPs and SSRs

Single nucleotide polymorphism (SNP) is the basis for studying pedigree genetic variation and a good material for molecular markers. In the arthropod *Anopheles gambiae*, multiple SNPs have been shown to be associated with chemical disease control agents (Weetman et al., 2010). SNP analysis was performed on all unigenes. SNP sites were screened according to the criterion of no more than three consecutive single base mismatches within 35 bp and quality depth (QD)>2.0. There were 169,913 and 158,460 SNP sites in each of the two female samples, the numbers of homozygous SNPs were 80,514 and 88,312, and the numbers of heterozygous SNPs were 89,399 and 70,148, respectively. The total number of SNPs in the male sample was 178,524, including 80,132 homoSNPs and 98,392 heteSNPs.

Microsatellites are tandemly repeated fragments, composed of 1–6 base pairs in length, also called simple repeat sequence (SSR). Its molecular marker function is similar to SNP. Because of rapid mutation rate and polymorphism, it is widely used in population genetics and phylogenetic identification (Fernandez-Silva et al., 2013; Sun et al., 2016). By analyzing transcriptome data, a total of 6,522 SSRs were obtained, which were composed of six types of SSRs. It included 3,232 mono-nucleotide repeat, 1 725 di-nucleotide repeat, 1,174 tri-nucleotide repeat, 69 tetra-nucleotide repeat, two penta-nucleotide repeat and two hexa-nucleotide repeat (Table S3). Mono-nucleotide repeat was the most important, accounting for 49.56%, which was consistent with previous research results (Qiu et al., 2017). SSR transcripts can identify homologous lineal genes in other species and facilitate the direct construction of maps in multiple species.

## Discussion

### Functional annotation of Nr database

It is not rare in insects that the matching rate with Nr database is not high. The annotation proportion of the *Ceracris kiangsu* Tsai (Li, Wang & Jiang, 2019) and *Tetrix japonica* (Qiu et al., 2017), was not more than 51%, and the annotation proportion of *Tettigonia chinensis* was only 17.72% (Han, Chang & Zhou, 2020). The same situation also exists in the research of other arthropods, like *Macrobrachium nipponense, Fenneropenaeus chinensis* and *Portunus trituberculatus* (Leu et al., 2011; Ma et al., 2012; Lv et al., 2014). This phenomenon can be caused by untranslated mRNA regions, non-conserved transcripts, new genes, and misassembled sequences (Mittapalli et al., 2010; Lv et al., 2014; Qiu et al., 2017).

### Candidate genes involved in pigment

Pteridine, melanin, and ommochrome regulate the generation of pigment patterns (Connahs, Rhen & Simmons, 2016). Ommochrome was associated with the synthesis of red, brown and yellow pigments, which are found in the eyes, epidermis, wings and other organs of insects. The function of ommochrome was related to tryptophan metabolism and can remove excessive tryptophan metabolites to regulate the production of pigments (Reed & Nagy, 2005). Bab was inhibited in males of *D. melanogaster*, which was reflected by two more black pigmentation at the tail end of males. In male *D. melanogaster*, HOX protein Abdominal-B inhibited bab expression. However, the specific expression of doublesex in females counteracts this inhibitory mechanism and can normally express bab (Wittkopp, Carroll & Kopp, 2003). Dopamine plays an important role in melanisation and sclerotisation, and also affects immune function (Verlinden, 2018). The black stripes are regulated by the T-box transcription factor Omb. Omb is controlled by Hh signaling pathway. Dpp signal pathway combines wingless and epidermal growth factor receiver signaling to regulate the width and spatial spatial cues of patterns and stripes (Wittkopp, Carroll & Kopp, 2003). The regulatory effect of segmentation is also highlighted in the individual immunity of insects. Previous studies have shown that segmentation plays different roles in innate immunity, wound healing and parasite resistance. The presence of pigment on the body surface of pathogen invasion proves that innate immunity is associated with pigmentation (Christensen et al., 2005). Pigmentation of the body surface can develop after injury and help wound healing (Galko & Krasnow, 2004). Experimental studies have shown that pigmentation enhances physical barriers and reduces parasite invasion (Dombeck & Jaenike, 2004). Pigmentation plays a role in intraspecific and interspecific communication and regulates physiological activities, such as body temperature, anti-drying, and light resistance (Clusella-Trullas & Nielsen, 2020). A variety of group behaviors have also been confirmed to be related to segmentation, including courtship (Yeh, Liou & True, 2006), foraging (Forsman et al., 2002) and social living (Sword et al., 2000).

### Immunity genes and pathways

CTL, also known as immulectin, can not only participate in innate immunity, but also recognize pathogenic microorganisms and activate specific immunity (Theopold et al., 1999; Koizumi et al., 1999), enhanced hemocyte encapsulation, melanization and enhanced phagocytic efficiency (Kawasaki, Kawasaki & Yamashina, 1989; Yu et al., 2006). Sod plays a key role in resisting virus and bacterial invasion (Shi et al., 2021), which can regulate the effect of cellular oxidative stress and participate in fat body immunity (Nojima et al., 2015). Peptidoglycan recognition proteins (PGRPs) are a family that recognize peptidoglycan and participate in immune response, including pgrp-sa, pgrp-sb, pgrp-sc, pgrp-sd, pgrp-lc and pgrp-le (Kurata, 2014). They are expressed in a variety of immune tissues, including blood cells, fat body, epidermis, gut, *etc*, and are responsible for recognizing bacterial invasion (Han et al., 2017), enhancing antimicrobial peptide secretion and bacterial phagocytosis (Goto et al., 2010). Apolipophorin includes three types, apolipophorin I (apoLp-I), apolipophorin II (apoLp-II) and apolipophorin III (apoLp-III). apoLp-III is a hemolymph lipid transporter (Weers & Ryan, 2006) that enhances phagocytic, antibacterial, and bacteriolytic abilities (Maravilla et al., 2020). ApoLp-I and apoLp-II are responsible for the transport and deposition of stratum corneum lipids to maintaining the barrier function of the stratum corneum (Zhao et al., 2020). Toll-LIKE receptor signaling detects bacterial infection and induces the production of antimicrobial peptides (Valanne, Wang & Rämet, 2011).

JAK-STAT signaling pathway is an important immune pathway that directly activates immune genes for immune resistance and stress response (Boutros, Agaisse & Perrimon, 2002; Agaisse & Perrimon, 2004). It can also regulate the expression of a variety of immune proteins, such as cytokines and stress response Tot protein (Kallio et al., 2010). JAK-STAT signaling pathway has been shown to function in the proliferation of blood cells, wound repair, and intestinal antimicrobial defense (Bangi, 2013; Myllymäki & Rämet, 2014). In addition to immune regulation, JAK-STAT signaling plays an important role in growth and development, regulating embryonic, wing, eye formation and stem cell maintenance (Bach et al., 2003; Wang et al., 2011). Wnt signaling pathway regulates multiple Wnt protein family members, such as receptor related protein (LDL), frizzled, dishevelled (Dsh/Dvl), *β*-catenin, glycogen synthase kinase-3 *β* (GSK-3 *β*), axin/conductin, adenomatous polyposis coli (APC) (Nusse et al., 1991). It has been widely studied in the fields of human immunity and cancer treatment (Steinhart & Angers, 2018). Wnt signaling pathway is related to insect wing development (Nüsslein-Volhard & Wieschaus, 1980), growth zone metabolism and cell division (Oberhofer et al., 2014).

### Insecticide resistance genes and pathways

Pesticides such as phenylpyrazole fipronil, isoxazolines and meta diamides mainly target GABA signaling pathway and block chloride ion channel to achieve insecticidal effect (Zhao et al., 2014). GABA signaling pathway can regulate chloride channel switch, which is the target site of cyclodiene and pyrazole, bicyclophosphate insecticides. The RyR signaling pathway is a Ca^2+^-dependent signaling pathway that regulates muscle excitation and contraction. Two new chemotypes of diamide insecticides activate the ryanodine receptor and release Ca^2+^ indefinitely, leading to muscle relaxation, paralysis, the inability to maneuver for food and die (Sattelle, Cordova & Cheek, 2008; Dunn et al., 2022). P450 signaling pathway is an important detoxification pathway, which can metabolize exogenous substances such as pesticides and is also responsible for the metabolism of endogenous compounds (Lu, Song & Zeng, 2021). Although its detoxification mechanism has not been reported, it has been extensively validated in insecticide resistance experiments, such as *Depressaria pastinacella*, *D. melanogaster*, *Musca domestica*, *Helicoverpa armigera*, *Anopheles gambiae*, *Apis mellifera* (Mao et al., 2006; Wen et al., 2006; Mao et al., 2007). P450 can catalyze oxidation, reduction, dehydrogenation, hydrolysis and other reactions, and has favorable metabolic effects on organophosphorus, pyrethroid insecticides and some toxic plant metabolites (Berenbaum & Johnson, 2015; Alyokhin & Chen, 2017).

Car Es genes are expressed in insect adipose bodies and participate in resistance to organophosphate (OP) and pyrethroids (Cui et al., 2015; Feng, Li & Liu, 2018). GST genes resist pesticides by directly metabolizing harmful substances, sequestering chemicals, or responding to oxidative stress (Liu et al., 2015; Pavlidi, Vontas & Van Leeuwen, 2018). There are nAChRs, which express excitatory receptors in the central nervous system and recognize each other with nicotinic acetylcholine (ACh) to control cation channels (Ujváry, 1999; Tomizawa & Casida, 2003). nAChRs are targets of necrotoxins and chlorinated nicotinyl insecticides (Lee et al., 2004). DDT genes were screened to act on the sodium channel and prolong its opening time, resulting in negative potential enhancement exceeding the threshold, repeated discharge or nerve membrane depolarization (Zhorov & Dong, 2017).

### The connection between the pigment, immunity, insecticide resistance

With the annotation of *F. qinlingensis* transcriptome data, we found that some genes of male and female were differentially expressed in the existing data. The allocation of immune resources and energy resources requires weighing various activities and functions to promote animal breeding, growth and development. The immunological cost of life history is not only the distribution of nutrients and energy, but also the mating risk (predation risk and disease risk) associated with the external environment (Roff, 1992). Therefore, the choice of mating risk and immune defense may be the reason for the differential expression. It has been reported in insects that the secretion of some specific hormones leads to female bias of immunity. Male specific testosterone has immuno-suppressive effect. Phenoloxidase, which is highly expressed in females for oviposition, is a component of immune defense (Christensen et al., 2005; Bear & Monteiro, 2013).

Klasing & Leshchinsky (1999) proposed that fast-living species and slow-living species have different immune tendencies based on the connection between immunity and life history. Fast-living species with high reproduction rates, short development times and short life spans tended to invest more in non-specific immunity and inflammatory repair. Relatively speaking, slow-living species with lower reproduction rates, longer development times and higher adult survival rates pay more attention to specific immunity and stable immunity that can be maintained for a long time. Insect pigments provide non-specific immunity for wound healing and parasite resistance. The expression of pigments with sex differences may also influence the immune effect of insects.

Does sex really lead to differences? Some researchers believe that the value of male choice to sacrifice immune investment and increase mating success rate exceeds the cost of shortening life span. Females are in more need to boost immunity to prolong their life. Especially in the face of immune pressure from parasites and bacteria, the optimal choice is that the male’s immune defense will decrease with the increase of sexual selection (Rolff, 2002; Stoehr & Kokko, 2006). However, the reasons for the differences are diverse. Age, sex, day and night duration, physical contact, chemical pheromones and other factors may be influencing factors, and controversial opinions always exist (Christensen et al., 2005; Lee, 2006). There are also completely opposite conclusions in the analysis of different species and models, and some people believe that sex and immune defense are not related (Brock, Murdock & Martin, 2014; Kelly et al., 2018). Due to the limited data and the combination of various factors, the correlation between sex and immune defense of *F. qinlingensis* needs to be further studied with more extensive sampling and setting replications. With the expansion and validation of more datasets in the future, the relationship between sex and immune defense will be better understood.

##  Supplemental Information

10.7717/peerj.15123/supp-1 Supplemental Information 1 Specimen of *Formosatettix qinlingensis*

10.7717/peerj.15123/supp-2 Supplemental Information 2 KEGG pathway enrichment of DEUs

10.7717/peerj.15123/supp-3 Supplemental Information 3 Correlation plot of qRT-PCR results and RNA-Seq analyses

10.7717/peerj.15123/supp-4 Supplemental Information 4 qRT-PCR primers of *Formosatettix qinlingensis*

10.7717/peerj.15123/supp-5 Supplemental Information 5 *Formosatettix qinlingensis* transcriptome proteins and reciprocal BLAST hit (RBH) results for identification of pigment, immunity and insecticide resistance genes DEUs are marked in red.

10.7717/peerj.15123/supp-6 Supplemental Information 6 The six SSRs types of *Formosatettix qinlingensis.*

## Additional Information and Declarations

### DNA Deposition

The following information was supplied regarding the deposition of DNA sequences: The raw data of Formosatettix qinlingensis is available at NCBI: SRR19977122, SRR19977123, and SRR19977124.

### Data Availability

The following information was supplied regarding data availability: The sequences for *Formosatettix qinlingensis* is available at NCBI: PRJNA855627, SRR19977122, SRR19977123, and SRR19977124. The transcriptomes are available at NCBI: SAMN29499290, SAMN29499291 and SAMN29499292. The raw data are available at NCBI: SRR19977122, SRR19977123, and SRR19977124.
