# Supplementary figures and images for: Transcriptome of the pygmy grasshopper Formosatettix qinlingensis (Orthoptera: Tetrigidae)
Source: PeerJ. 2023 Mar 30;11:e15123. doi: 10.7717/peerj.15123 (PMC10066883; doi:10.7717/peerj.15123)

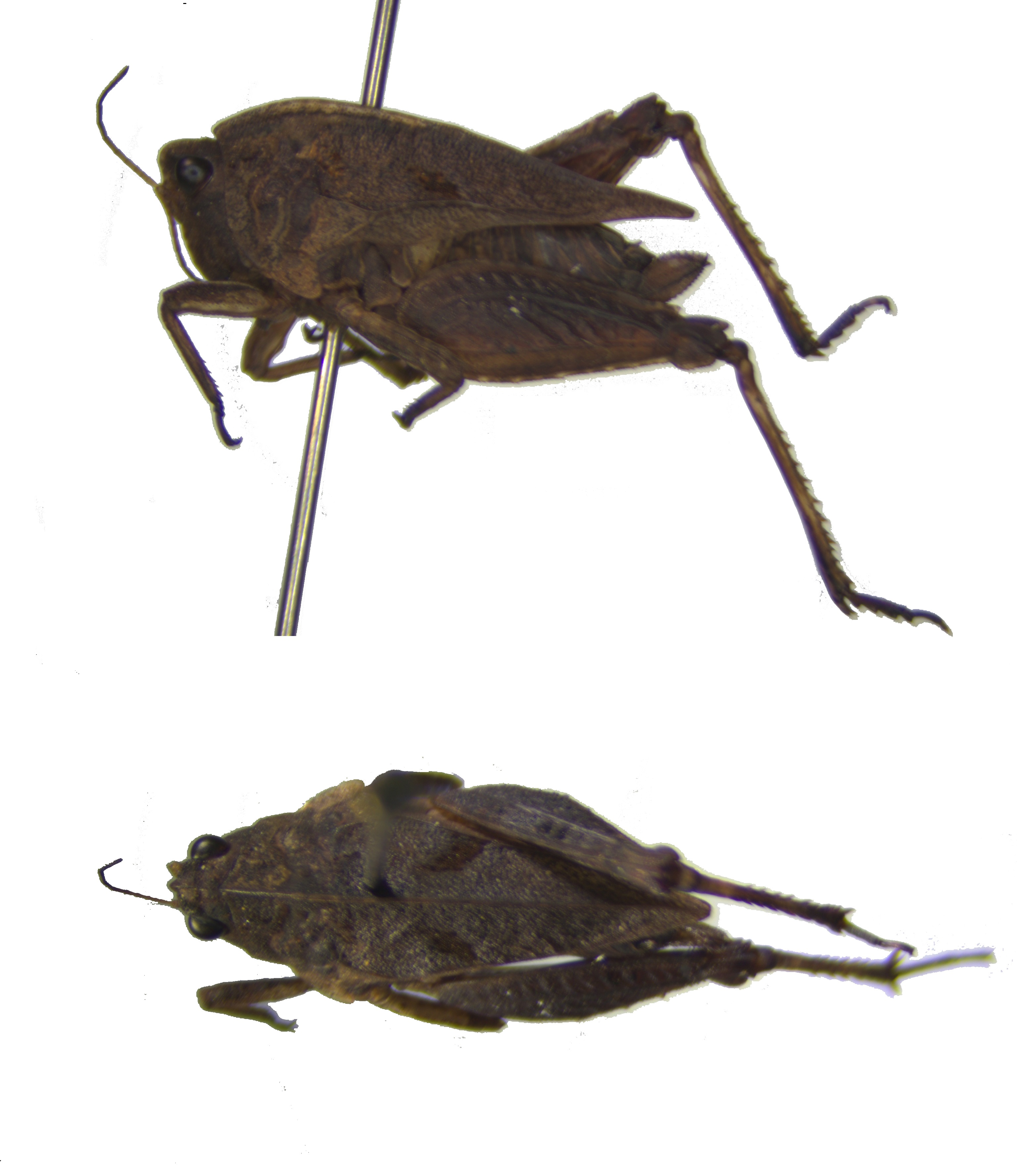

Supplement: Supplemental Information 1 [file peerj-11-15123-s001.png]

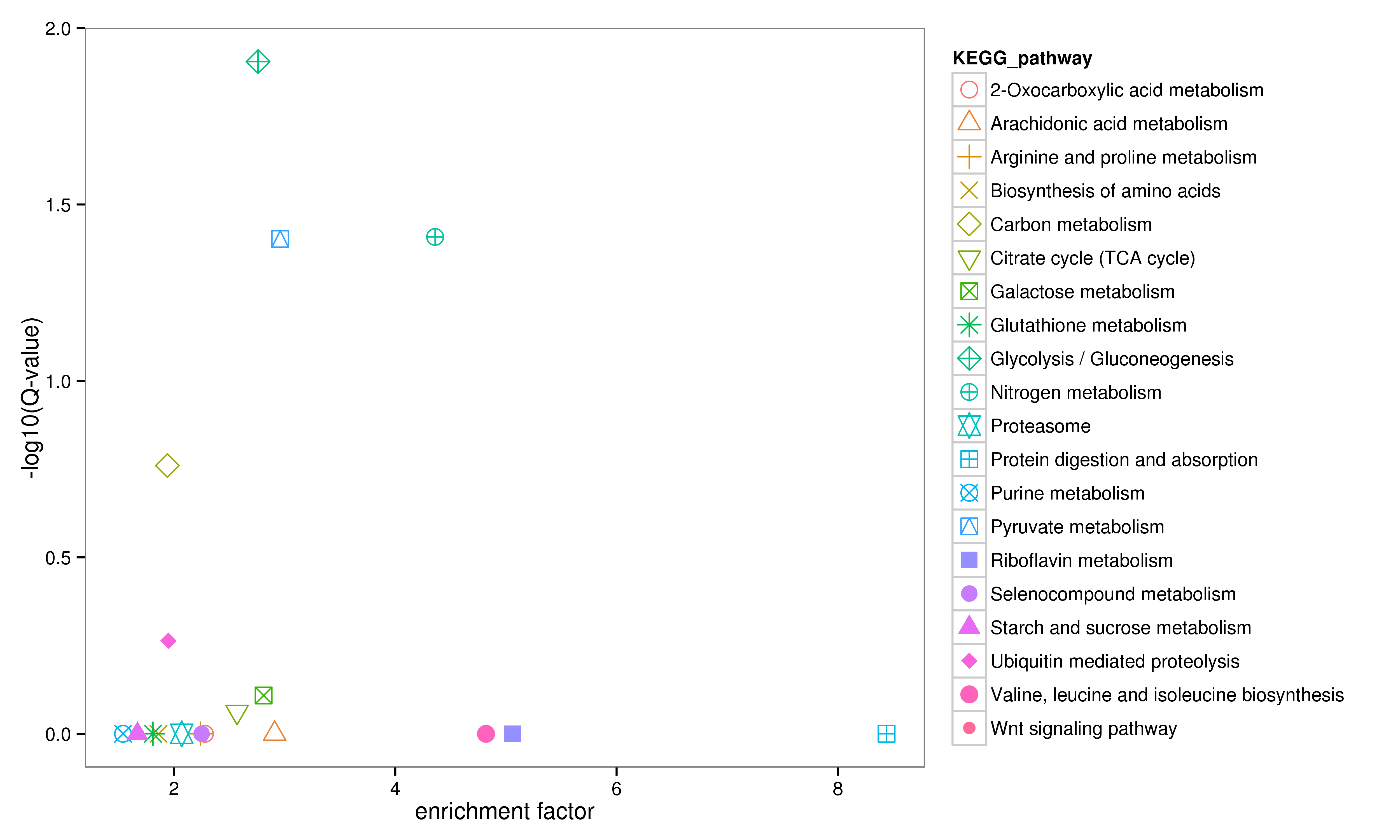

Supplement: Supplemental Information 2 [file peerj-11-15123-s002.png]

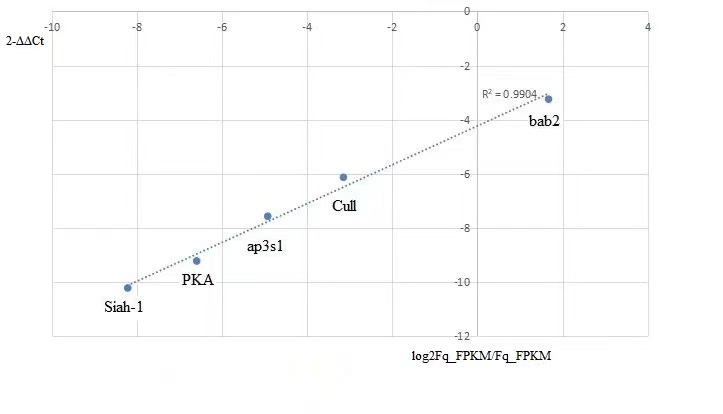

Supplement: Supplemental Information 3 [file peerj-11-15123-s003.jpg]
