# Supplementary material for: Transcriptome of the pygmy grasshopper Formosatettix qinlingensis (Orthoptera: Tetrigidae)
Source: PeerJ. 2023 Mar 30;11:e15123. doi: 10.7717/peerj.15123 (PMC10066883; doi:10.7717/peerj.15123)
Supplement: Supplemental Information 4 [file peerj-11-15123-s004.docx]

Table S1

| Primer | Sequence | | gene ID |
| --- | --- | --- | --- |
| PKA forward primers | | CAGGTCTTGTTGGCTCTC | Unigene_44709 |
| PKA reverse primers | | GTGAGGTCCGTGTTGATG |  |
| Siah-1 forward primers | | CAGGTCTTGTTGGCTCTC | Unigene_47083 |
| Siah-1 reverse primers | | GCTGTATGGCTCTGTTCA |  |
| Cull forward primers | | GGAGAGTTACGCCAGGTA | Unigene_31264 |
| Cull reverse primers | | TTCTCGTCGCAGTTCAAG |  |
| ap3s1 forward primers | | CATTGTTGACTTCTCTTCGT | Unigene_52233 |
| ap3s1 reverse primers | | TTACACTCGCATCCTTCG |  |
| bab2 forward primers | | ATCATCGCTGTCACTTGG | Unigene_06659 |
| bab2 reverse primers | | ACCTCTTGTGCTTGTTATTG |  |
| β-actin forward primers | | AGCCTCATCACCTACATAAC | Unigene_05368 |
| β-actin reverse primers | | CTTGTCCGTCTCCAGTTC |  |
