# Supplementary material for: Transcriptome of the pygmy grasshopper Formosatettix qinlingensis (Orthoptera: Tetrigidae)
Source: PeerJ. 2023 Mar 30;11:e15123. doi: 10.7717/peerj.15123 (PMC10066883; doi:10.7717/peerj.15123)
Supplement: Supplemental Information 6 [file peerj-11-15123-s006.docx]

Table S3 six types of SSRs of *Formosatettix qinlingensis*

| Type | Number |
| --- | --- |
| mono-nucleotide repeat | 3232 |
| di-nucleotide repeat | 1725 |
| tri-nucleotide repeat | 1174 |
| tetra-nucleotide repeat | 69 |
| penta-nucleotide repeat | 2 |
| hexa-nucleotide repeat | 2 |
| Total | 6522 |
